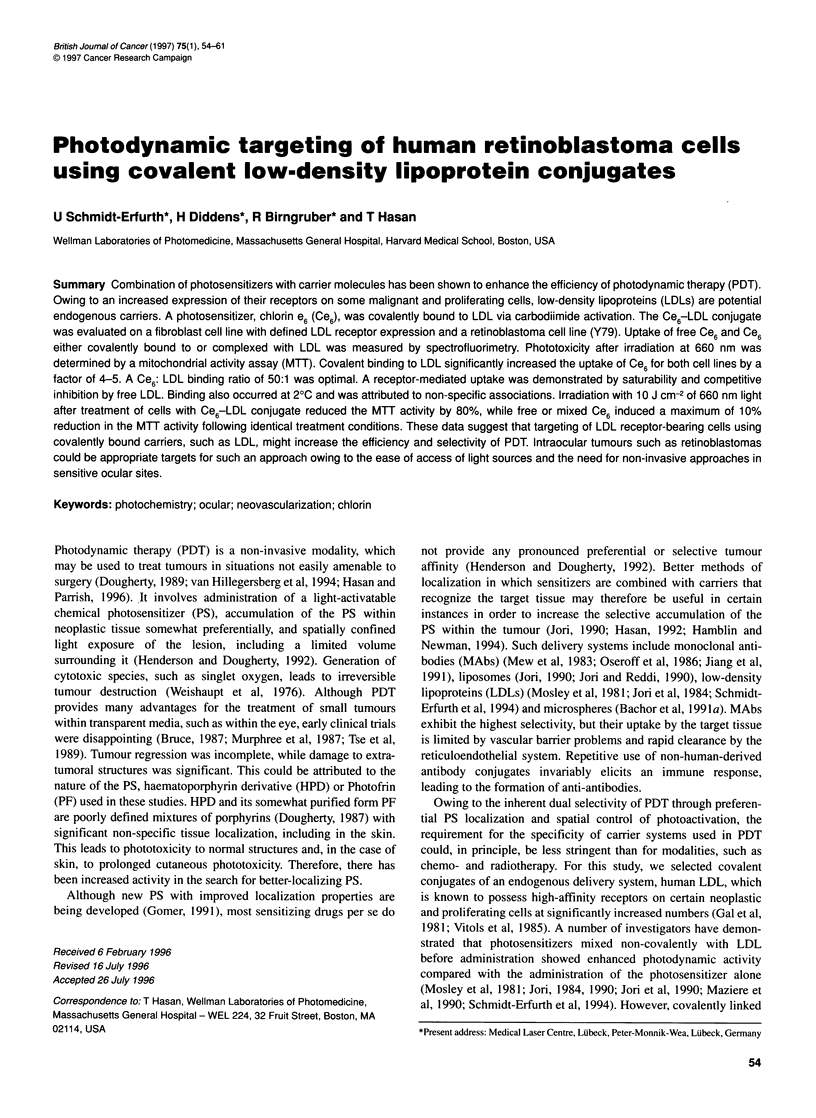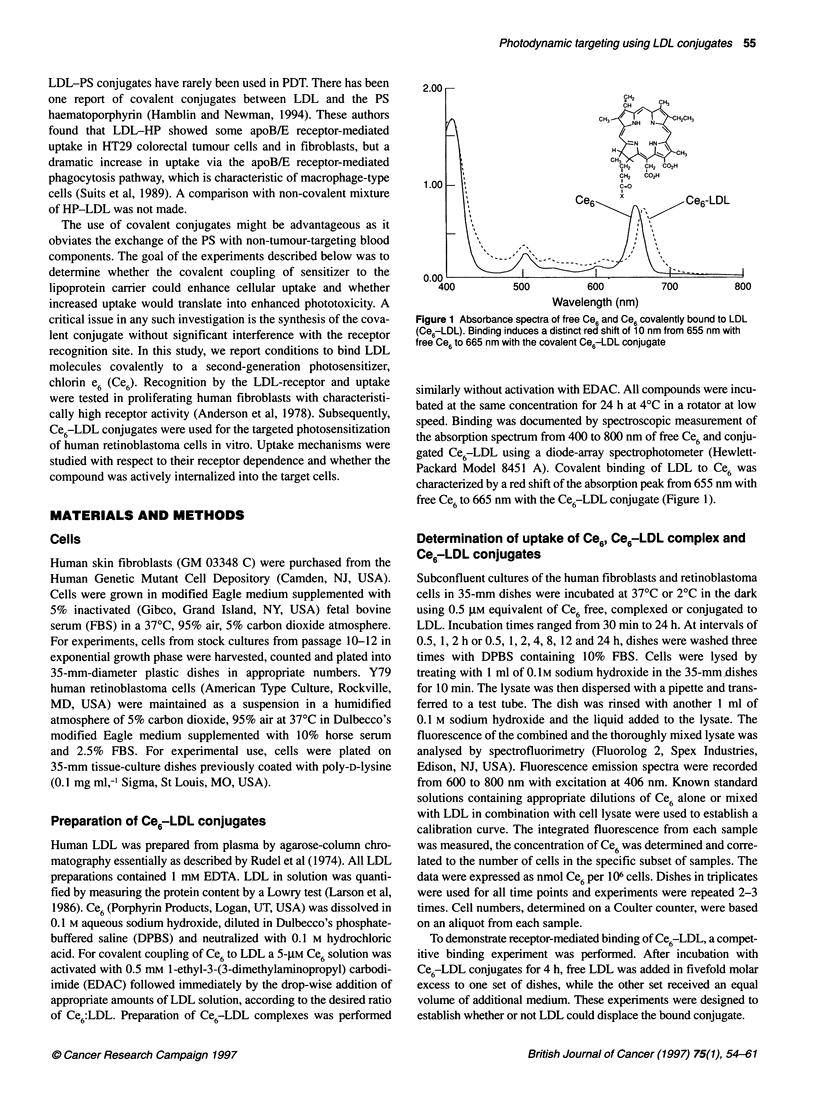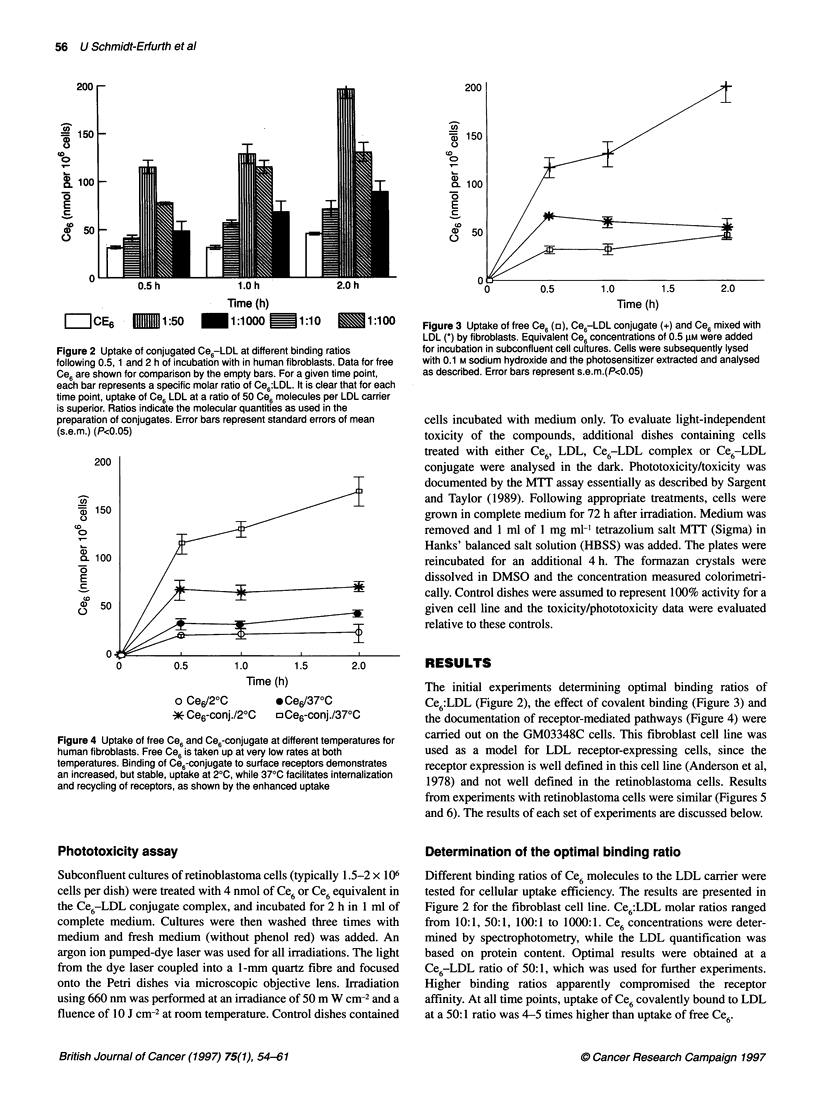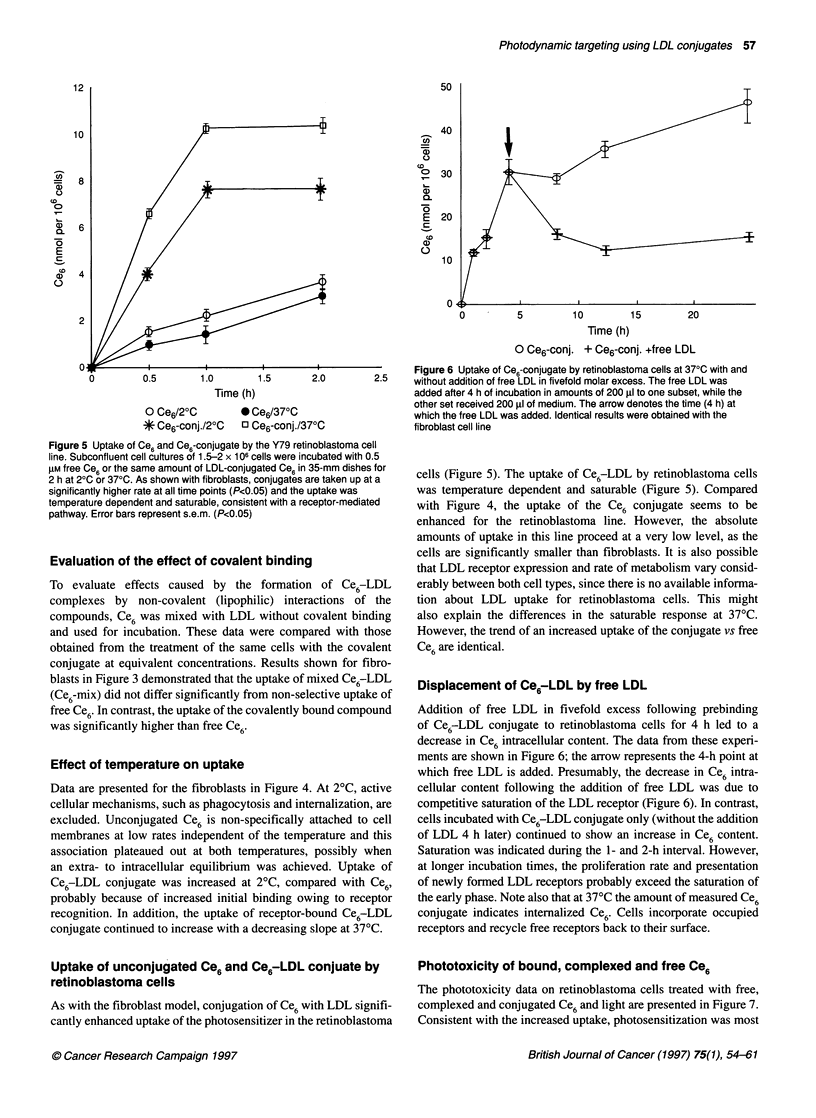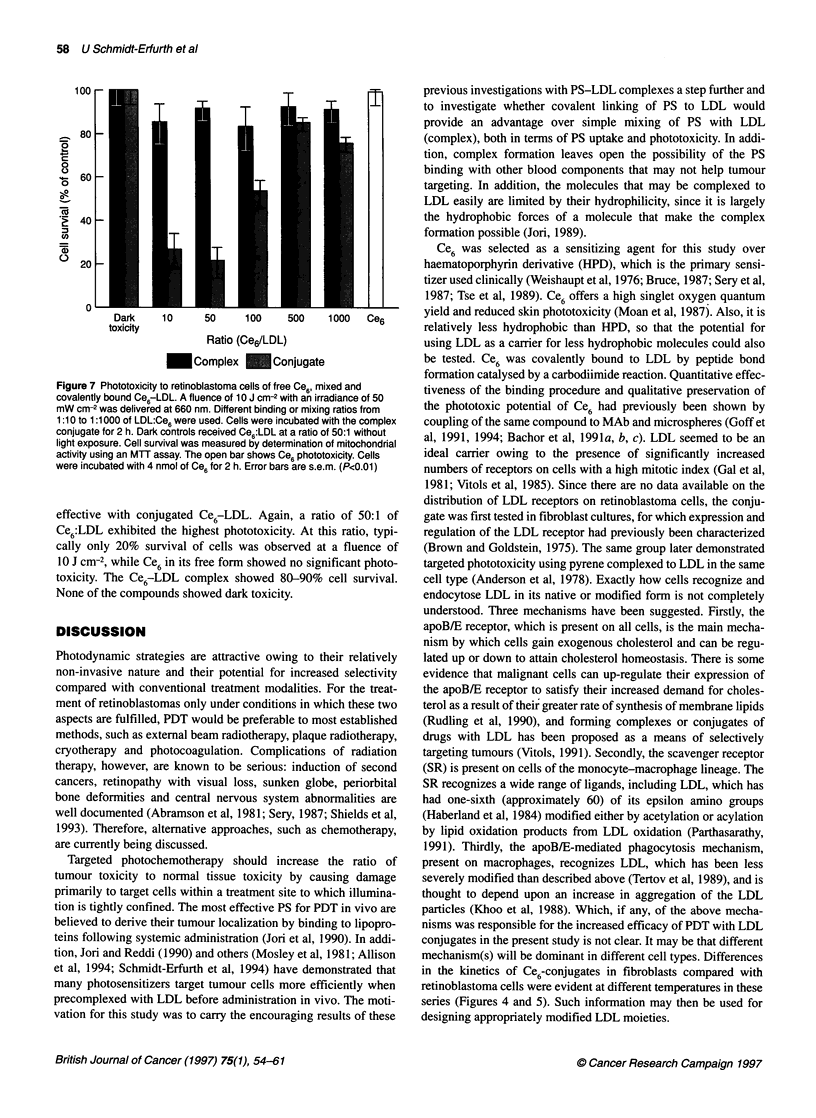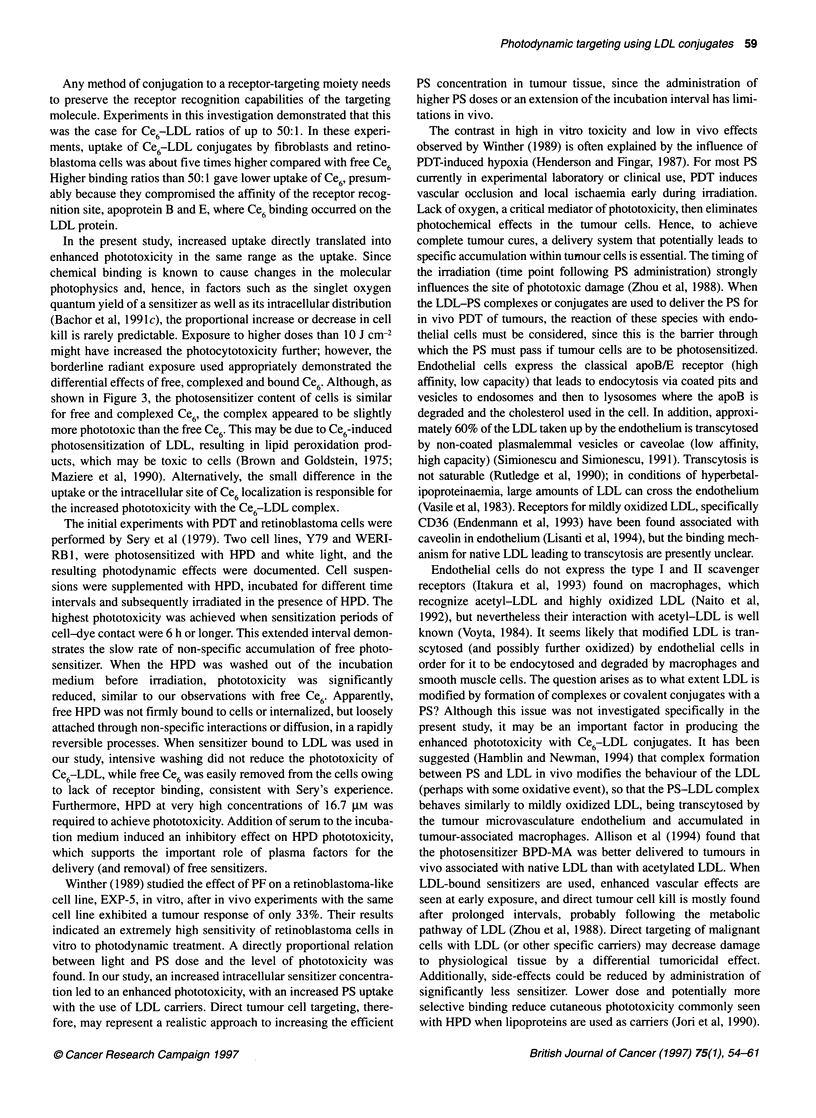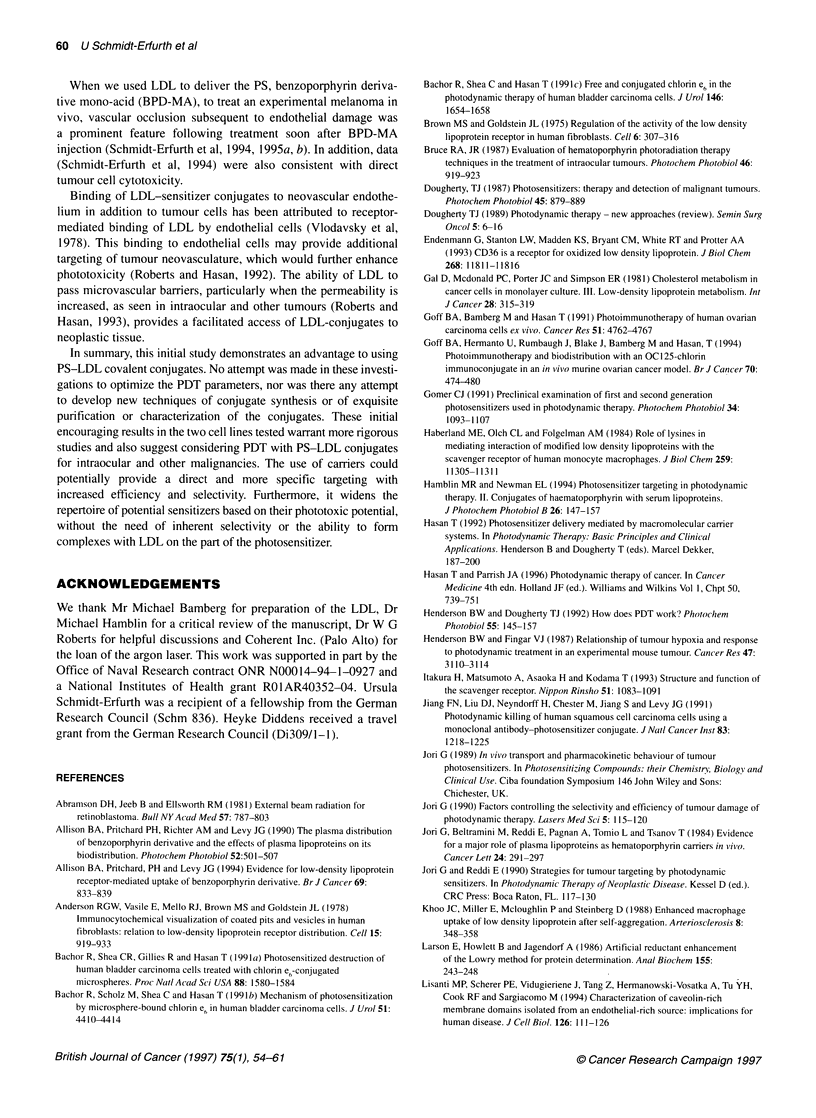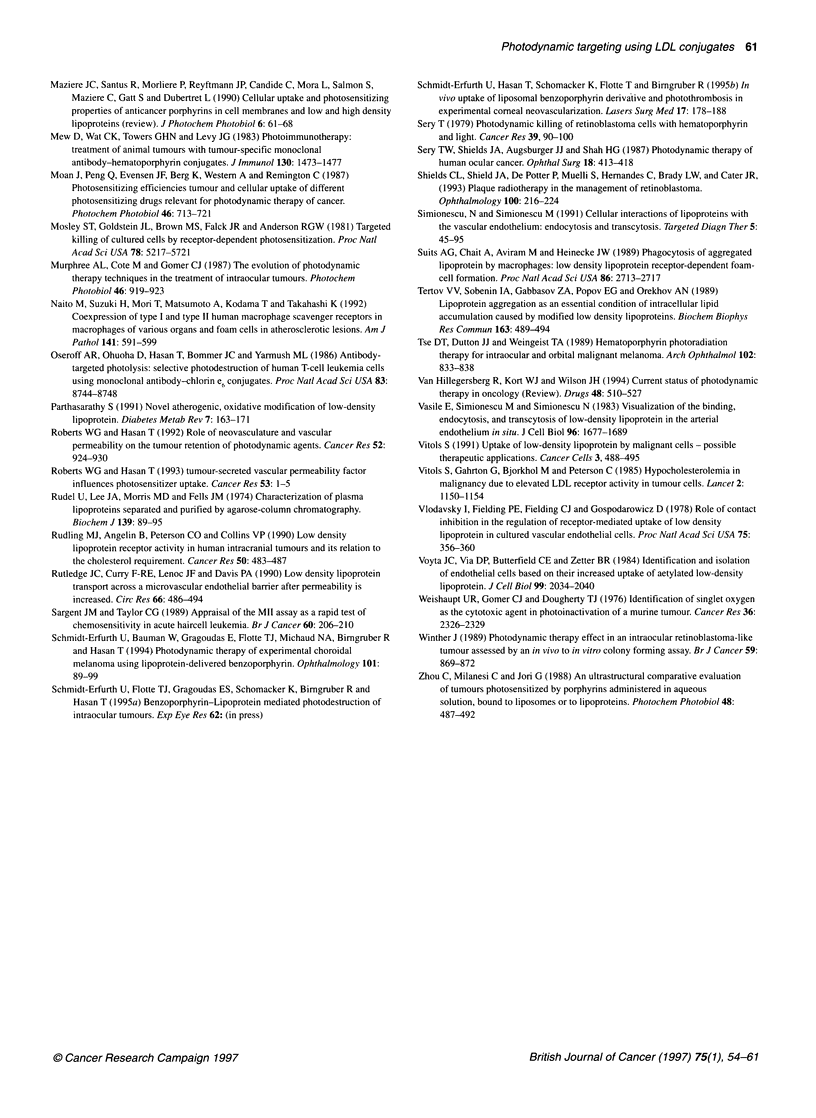# Photodynamic targeting of human retinoblastoma cells using covalent low-density lipoprotein conjugates.

**DOI:** 10.1038/bjc.1997.9

**Published:** 1997

**Authors:** U. Schmidt-Erfurth, H. Diddens, R. Birngruber, T. Hasan

**Affiliations:** Wellman Laboratories of Photomedicine, Massachusetts General Hospital, Harvard Medical School, Boston 02114, USA.

## Abstract

Combination of photosensitizers with carrier molecules has been shown to enhance the efficiency of photodynamic therapy (PDT). Owing to an increased expression of their receptors on some malignant and proliferating cells, low-density lipoproteins (LDLs) are potential endogenous carriers. A photosensitizer, chlorin e6 (Ce6), was covalently bound to LDL via carbodiimide activation. The Ce6-LDL conjugate was evaluated on a fibroblast cell line with defined LDL receptor expression and a retinoblastoma cell line (Y79). Uptake of free Ce6 and Ce6 either covalently bound to or complexed with LDL was measured by spectrofluorimetry. Phototoxicity after irradiation at 660 nm was determined by a mitochondrial activity assay (MTT). Covalent binding to LDL significantly increased the uptake of Ce6 for both cell lines by a factor of 4-5. A Ce6: LDL binding ratio of 50:1 was optimal. A receptor-mediated uptake was demonstrated by saturability and competitive inhibition by free LDL. Binding also occurred at 2 degrees C and was attributed to non-specific associations. Irradiation with 10 J cm-2 of 660 nm light after treatment of cells with Ce6-LDL conjugate reduced the MTT activity by 80%, while free or mixed Ce6 induced a maximum of 10% reduction in the MTT activity following identical treatment conditions. These data suggest that targeting of LDL receptor-bearing cells using covalently bound carriers, such as LDL, might increase the efficiency and selectivity of PDT. Intraocular tumours such as retinoblastomas could be appropriate targets for such an approach owing to the ease of access of light sources and the need for non-invasive approaches in sensitive ocular sites.


British Journal of Cancer (1997) 75(1), 54–61
© 1997 Cancer Research Campaign

### Photodynamic targeting of human retinoblastoma cells using covalent low-density lipoprotein conjugates

U Schmidt-Erfurth*, H Diddens*, R Birngruber* and T Hasan

Wellman Laboratories of Photomedicine, Massachusetts General Hospital, Harvard Medical School, Boston, USA

**Summary** Combination of photosensitizers with carrier molecules has been shown to enhance the efficiency of photodynamic therapy (PDT). Owing to an increased expression of their receptors on some malignant and proliferating cells, low-density lipoproteins (LDLs) are potential endogenous carriers. A photosensitizer, chlorin $e_6$ ($Ce_6$), was covalently bound to LDL via carbodiimide activation. The $Ce_6$–LDL conjugate was evaluated on a fibroblast cell line with defined LDL receptor expression and a retinoblastoma cell line (Y79). Uptake of free $Ce_6$ and $Ce_6$ either covalently bound to or complexed with LDL was measured by spectrofluorimetry. Phototoxicity after irradiation at 660 nm was determined by a mitochondrial activity assay (MTT). Covalent binding to LDL significantly increased the uptake of $Ce_6$ for both cell lines by a factor of 4–5. A $Ce_6$: LDL binding ratio of 50:1 was optimal. A receptor-mediated uptake was demonstrated by saturability and competitive inhibition by free LDL. Binding also occurred at 2°C and was attributed to non-specific associations. Irradiation with 10 J $cm^{-2}$ of 660 nm light after treatment of cells with $Ce_6$–LDL conjugate reduced the MTT activity by 80%, while free or mixed $Ce_6$ induced a maximum of 10% reduction in the MTT activity following identical treatment conditions. These data suggest that targeting of LDL receptor-bearing cells using covalently bound carriers, such as LDL, might increase the efficiency and selectivity of PDT. Intraocular tumours such as retinoblastomas could be appropriate targets for such an approach owing to the ease of access of light sources and the need for non-invasive approaches in sensitive ocular sites.

**Keywords:** photochemistry; ocular; neovascularization; chlorin

Photodynamic therapy (PDT) is a non-invasive modality, which may be used to treat tumours in situations not easily amenable to surgery (Dougherty, 1989; van Hillegersberg et al, 1994; Hasan and Parrish, 1996). It involves administration of a light-activatable chemical photosensitizer (PS), accumulation of the PS within neoplastic tissue somewhat preferentially, and spatially confined light exposure of the lesion, including a limited volume surrounding it (Henderson and Dougherty, 1992). Generation of cytotoxic species, such as singlet oxygen, leads to irreversible tumour destruction (Weishaupt et al, 1976). Although PDT provides many advantages for the treatment of small tumours within transparent media, such as within the eye, early clinical trials were disappointing (Bruce, 1987; Murphree et al, 1987; Tse et al, 1989). Tumour regression was incomplete, while damage to extra-tumoral structures was significant. This could be attributed to the nature of the PS, haematoporphyrin derivative (HPD) or Photofrin (PF) used in these studies. HPD and its somewhat purified form PF are poorly defined mixtures of porphyrins (Dougherty, 1987) with significant non-specific tissue localization, including in the skin. This leads to phototoxicity to normal structures and, in the case of skin, to prolonged cutaneous phototoxicity. Therefore, there has been increased activity in the search for better-localizing PS.

Although new PS with improved localization properties are being developed (Gomer, 1991), most sensitizing drugs per se do not provide any pronounced preferential or selective tumour affinity (Henderson and Dougherty, 1992). Better methods of localization in which sensitizers are combined with carriers that recognize the target tissue may therefore be useful in certain instances in order to increase the selective accumulation of the PS within the tumour (Jori, 1990; Hasan, 1992; Hamblin and Newman, 1994). Such delivery systems include monoclonal antibodies (MAbs) (Mew et al, 1983; Oseroff et al, 1986; Jiang et al, 1991), liposomes (Jori, 1990; Jori and Reddi, 1990), low-density lipoproteins (LDLs) (Mosley et al, 1981; Jori et al, 1984; Schmidt-Erfurth et al, 1994) and microspheres (Bachor et al, 1991*a*). MAbs exhibit the highest selectivity, but their uptake by the target tissue is limited by vascular barrier problems and rapid clearance by the reticuloendothelial system. Repetitive use of non-human-derived antibody conjugates invariably elicits an immune response, leading to the formation of anti-antibodies.

Owing to the inherent dual selectivity of PDT through preferential PS localization and spatial control of photoactivation, the requirement for the specificity of carrier systems used in PDT could, in principle, be less stringent than for modalities, such as chemo- and radiotherapy. For this study, we selected covalent conjugates of an endogenous delivery system, human LDL, which is known to possess high-affinity receptors on certain neoplastic and proliferating cells at significantly increased numbers (Gal et al, 1981; Vitols et al, 1985). A number of investigators have demonstrated that photosensitizers mixed non-covalently with LDL before administration showed enhanced photodynamic activity compared with the administration of the photosensitizer alone (Mosley et al, 1981; Jori, 1984, 1990; Jori et al, 1990; Maziere et al, 1990; Schmidt-Erfurth et al, 1994). However, covalently linked

*Received 6 February 1996*
*Revised 16 July 1996*
*Accepted 26 July 1996*

*Correspondence to:* T Hasan, Wellman Laboratories of Photomedicine, Massachusetts General Hospital – WEL 224, 32 Fruit Street, Boston, MA 02114, USA

*Present address: Medical Laser Centre, Lübeck, Peter-Monnik-Wea, Lübeck, Germany

LDL–PS conjugates have rarely been used in PDT. There has been one report of covalent conjugates between LDL and the PS haematoporphyrin (Hamblin and Newman, 1994). These authors found that LDL–HP showed some apoB/E receptor-mediated uptake in HT29 colorectal tumour cells and in fibroblasts, but a dramatic increase in uptake via the apoB/E receptor-mediated phagocytosis pathway, which is characteristic of macrophage-type cells (Suits et al, 1989). A comparison with non-covalent mixture of HP–LDL was not made.

The use of covalent conjugates might be advantageous as it obviates the exchange of the PS with non-tumour-targeting blood components. The goal of the experiments described below was to determine whether the covalent coupling of sensitizer to the lipoprotein carrier could enhance cellular uptake and whether increased uptake would translate into enhanced phototoxicity. A critical issue in any such investigation is the synthesis of the covalent conjugate without significant interference with the receptor recognition site. In this study, we report conditions to bind LDL molecules covalently to a second-generation photosensitizer, chlorin $e_6$ ($Ce_6$). Recognition by the LDL-receptor and uptake were tested in proliferating human fibroblasts with characteristically high receptor activity (Anderson et al, 1978). Subsequently, $Ce_6$–LDL conjugates were used for the targeted photosensitization of human retinoblastoma cells in vitro. Uptake mechanisms were studied with respect to their receptor dependence and whether the compound was actively internalized into the target cells.

### MATERIALS AND METHODS

### Cells

Human skin fibroblasts (GM 03348 C) were purchased from the Human Genetic Mutant Cell Depository (Camden, NJ, USA). Cells were grown in modified Eagle medium supplemented with 5% inactivated (Gibco, Grand Island, NY, USA) fetal bovine serum (FBS) in a 37°C, 95% air, 5% carbon dioxide atmosphere. For experiments, cells from stock cultures from passage 10–12 in exponential growth phase were harvested, counted and plated into 35-mm-diameter plastic dishes in appropriate numbers. Y79 human retinoblastoma cells (American Type Culture, Rockville, MD, USA) were maintained as a suspension in a humidified atmosphere of 5% carbon dioxide, 95% air at 37°C in Dulbecco's modified Eagle medium supplemented with 10% horse serum and 2.5% FBS. For experimental use, cells were plated on 35-mm tissue-culture dishes previously coated with poly-D-lysine (0.1 mg $ml^{-1}$, Sigma, St Louis, MO, USA).

### Preparation of $Ce_6$–LDL conjugates

Human LDL was prepared from plasma by agarose-column chromatography essentially as described by Rudel et al (1974). All LDL preparations contained 1 mM EDTA. LDL in solution was quantified by measuring the protein content by a Lowry test (Larson et al, 1986). $Ce_6$ (Porphyrin Products, Logan, UT, USA) was dissolved in 0.1 M aqueous sodium hydroxide, diluted in Dulbecco's phosphate-buffered saline (DPBS) and neutralized with 0.1 M hydrochloric acid. For covalent coupling of $Ce_6$ to LDL a 5-μM $Ce_6$ solution was activated with 0.5 mM 1-ethyl-3-(3-dimethylaminopropyl) carbodiimide (EDAC) followed immediately by the drop-wise addition of appropriate amounts of LDL solution, according to the desired ratio of $Ce_6$:LDL. Preparation of $Ce_6$–LDL complexes was performed

**Figure 1** Absorbance spectra of free $Ce_6$ and $Ce_6$ covalently bound to LDL ($Ce_6$–LDL). Binding induces a distinct red shift of 10 nm from 655 nm with free $Ce_6$ to 665 nm with the covalent $Ce_6$–LDL conjugate similarly without activation with EDAC. All compounds were incubated at the same concentration for 24 h at 4°C in a rotator at low speed. Binding was documented by spectroscopic measurement of the absorption spectrum from 400 to 800 nm of free $Ce_6$ and conjugated $Ce_6$–LDL using a diode-array spectrophotometer (Hewlett-Packard Model 8451 A). Covalent binding of LDL to $Ce_6$ was characterized by a red shift of the absorption peak from 655 nm with free $Ce_6$ to 665 nm with the $Ce_6$–LDL conjugate (Figure 1).

### Determination of uptake of $Ce_6$, $Ce_6$–LDL complex and $Ce_6$–LDL conjugates

Subconfluent cultures of the human fibroblasts and retinoblastoma cells in 35-mm dishes were incubated at 37°C or 2°C in the dark using 0.5 μM equivalent of $Ce_6$ free, complexed or conjugated to LDL. Incubation times ranged from 30 min to 24 h. At intervals of 0.5, 1, 2 h or 0.5, 1, 2, 4, 8, 12 and 24 h, dishes were washed three times with DPBS containing 10% FBS. Cells were lysed by treating with 1 ml of 0.1M sodium hydroxide in the 35-mm dishes for 10 min. The lysate was then dispersed with a pipette and transferred to a test tube. The dish was rinsed with another 1 ml of 0.1 M sodium hydroxide and the liquid added to the lysate. The fluorescence of the combined and the thoroughly mixed lysate was analysed by spectrofluorimetry (Fluorolog 2, Spex Industries, Edison, NJ, USA). Fluorescence emission spectra were recorded from 600 to 800 nm with excitation at 406 nm. Known standard solutions containing appropriate dilutions of $Ce_6$ alone or mixed with LDL in combination with cell lysate were used to establish a calibration curve. The integrated fluorescence from each sample was measured, the concentration of $Ce_6$ was determined and correlated to the number of cells in the specific subset of samples. The data were expressed as nmol $Ce_6$ per $10^6$ cells. Dishes in triplicates were used for all time points and experiments were repeated 2–3 times. Cell numbers, determined on a Coulter counter, were based on an aliquot from each sample.

To demonstrate receptor-mediated binding of $Ce_6$–LDL, a competitive binding experiment was performed. After incubation with $Ce_6$–LDL conjugates for 4 h, free LDL was added in fivefold molar excess to one set of dishes, while the other set received an equal volume of additional medium. These experiments were designed to establish whether or not LDL could displace the bound conjugate.

*© Cancer Research Campaign 1997*

**Figure 2** Uptake of conjugated $Ce_6$–LDL at different binding ratios following 0.5, 1 and 2 h of incubation with in human fibroblasts. Data for free $Ce_6$ are shown for comparison by the empty bars. For a given time point, each bar represents a specific molar ratio of $Ce_6$:LDL. It is clear that for each time point, uptake of $Ce_6$ LDL at a ratio of 50 $Ce_6$ molecules per LDL carrier is superior. Ratios indicate the molecular quantities as used in the preparation of conjugates. Error bars represent standard errors of mean (s.e.m.) ($P$<0.05)

**Figure 4** Uptake of free $Ce_6$ and $Ce_6$-conjugate at different temperatures for human fibroblasts. Free $Ce_6$ is taken up at very low rates at both temperatures. Binding of $Ce_6$-conjugate to surface receptors demonstrates an increased, but stable, uptake at 2°C, while 37°C facilitates internalization and recycling of receptors, as shown by the enhanced uptake

### Phototoxicity assay

Subconfluent cultures of retinoblastoma cells (typically 1.5–2 × $10^6$ cells per dish) were treated with 4 nmol of $Ce_6$ or $Ce_6$ equivalent in the $Ce_6$–LDL conjugate complex, and incubated for 2 h in 1 ml of complete medium. Cultures were then washed three times with medium and fresh medium (without phenol red) was added. An argon ion pumped-dye laser was used for all irradiations. The light from the dye laser coupled into a 1-mm quartz fibre and focused onto the Petri dishes via microscopic objective lens. Irradiation using 660 nm was performed at an irradiance of 50 m W $cm^{-2}$ and a fluence of 10 J $cm^{-2}$ at room temperature. Control dishes contained

**Figure 3** Uptake of free $Ce_6$ (□), $Ce_6$–LDL conjugate (+) and $Ce_6$ mixed with LDL (*) by fibroblasts. Equivalent $Ce_6$ concentrations of 0.5 μM were added for incubation in subconfluent cell cultures. Cells were subsequently lysed with 0.1 M sodium hydroxide and the photosensitizer extracted and analysed as described. Error bars represent s.e.m.($P$<0.05)

cells incubated with medium only. To evaluate light-independent toxicity of the compounds, additional dishes containing cells treated with either $Ce_6$, LDL, $Ce_6$–LDL complex or $Ce_6$–LDL conjugate were analysed in the dark. Phototoxicity/toxicity was documented by the MTT assay essentially as described by Sargent and Taylor (1989). Following appropriate treatments, cells were grown in complete medium for 72 h after irradiation. Medium was removed and 1 ml of 1 mg $ml^{-1}$ tetrazolium salt MTT (Sigma) in Hanks' balanced salt solution (HBSS) was added. The plates were reincubated for an additional 4 h. The formazan crystals were dissolved in DMSO and the concentration measured colorimetrically. Control dishes were assumed to represent 100% activity for a given cell line and the toxicity/phototoxicity data were evaluated relative to these controls.

### RESULTS

The initial experiments determining optimal binding ratios of $Ce_6$:LDL (Figure 2), the effect of covalent binding (Figure 3) and the documentation of receptor-mediated pathways (Figure 4) were carried out on the GM03348C cells. This fibroblast cell line was used as a model for LDL receptor-expressing cells, since the receptor expression is well defined in this cell line (Anderson et al, 1978) and not well defined in the retinoblastoma cells. Results from experiments with retinoblastoma cells were similar (Figures 5 and 6). The results of each set of experiments are discussed below.

### Determination of the optimal binding ratio

Different binding ratios of $Ce_6$ molecules to the LDL carrier were tested for cellular uptake efficiency. The results are presented in Figure 2 for the fibroblast cell line. $Ce_6$:LDL molar ratios ranged from 10:1, 50:1, 100:1 to 1000:1. $Ce_6$ concentrations were determined by spectrophotometry, while the LDL quantification was based on protein content. Optimal results were obtained at a $Ce_6$–LDL ratio of 50:1, which was used for further experiments. Higher binding ratios apparently compromised the receptor affinity. At all time points, uptake of $Ce_6$ covalently bound to LDL at a 50:1 ratio was 4–5 times higher than uptake of free $Ce_6$.

© Cancer Research Campaign 1997

**Figure 5** Uptake of $Ce_6$ and $Ce_6$-conjugate by the Y79 retinoblastoma cell line. Subconfluent cell cultures of 1.5–2 × $10^6$ cells were incubated with 0.5 μM free $Ce_6$ or the same amount of LDL-conjugated $Ce_6$ in 35-mm dishes for 2 h at 2°C or 37°C. As shown with fibroblasts, conjugates are taken up at a significantly higher rate at all time points ($P$<0.05) and the uptake was temperature dependent and saturable, consistent with a receptor-mediated pathway. Error bars represent s.e.m. ($P$<0.05)

### Evaluation of the effect of covalent binding

To evaluate effects caused by the formation of $Ce_6$–LDL complexes by non-covalent (lipophilic) interactions of the compounds, $Ce_6$ was mixed with LDL without covalent binding and used for incubation. These data were compared with those obtained from the treatment of the same cells with the covalent conjugate at equivalent concentrations. Results shown for fibroblasts in Figure 3 demonstrated that the uptake of mixed $Ce_6$–LDL ($Ce_6$-mix) did not differ significantly from non-selective uptake of free $Ce_6$. In contrast, the uptake of the covalently bound compound was significantly higher than free $Ce_6$.

### Effect of temperature on uptake

Data are presented for the fibroblasts in Figure 4. At 2°C, active cellular mechanisms, such as phagocytosis and internalization, are excluded. Unconjugated $Ce_6$ is non-specifically attached to cell membranes at low rates independent of the temperature and this association plateaued out at both temperatures, possibly when an extra- to intracellular equilibrium was achieved. Uptake of $Ce_6$–LDL conjugate was increased at 2°C, compared with $Ce_6$, probably because of increased initial binding owing to receptor recognition. In addition, the uptake of receptor-bound $Ce_6$–LDL conjugate continued to increase with a decreasing slope at 37°C.

### Uptake of unconjugated $Ce_6$ and $Ce_6$–LDL conjuate by retinoblastoma cells

As with the fibroblast model, conjugation of $Ce_6$ with LDL significantly enhanced uptake of the photosensitizer in the retinoblastoma cells (Figure 5). The uptake of $Ce_6$–LDL by retinoblastoma cells was temperature dependent and saturable (Figure 5). Compared with Figure 4, the uptake of the $Ce_6$ conjugate seems to be enhanced for the retinoblastoma line. However, the absolute amounts of uptake in this line proceed at a very low level, as the cells are significantly smaller than fibroblasts. It is also possible that LDL receptor expression and rate of metabolism vary considerably between both cell types, since there is no available information about LDL uptake for retinoblastoma cells. This might also explain the differences in the saturable response at 37°C. However, the trend of an increased uptake of the conjugate *vs* free $Ce_6$ are identical.

**Figure 6** Uptake of $Ce_6$-conjugate by retinoblastoma cells at 37°C with and without addition of free LDL in fivefold molar excess. The free LDL was added after 4 h of incubation in amounts of 200 μl to one subset, while the other set received 200 μl of medium. The arrow denotes the time (4 h) at which the free LDL was added. Identical results were obtained with the fibroblast cell line

### Displacement of $Ce_6$–LDL by free LDL

Addition of free LDL in fivefold excess following prebinding of $Ce_6$–LDL conjugate to retinoblastoma cells for 4 h led to a decrease in $Ce_6$ intracellular content. The data from these experiments are shown in Figure 6; the arrow represents the 4-h point at which free LDL is added. Presumably, the decrease in $Ce_6$ intracellular content following the addition of free LDL was due to competitive saturation of the LDL receptor (Figure 6). In contrast, cells incubated with $Ce_6$–LDL conjugate only (without the addition of LDL 4 h later) continued to show an increase in $Ce_6$ content. Saturation was indicated during the 1- and 2-h interval. However, at longer incubation times, the proliferation rate and presentation of newly formed LDL receptors probably exceed the saturation of the early phase. Note also that at 37°C the amount of measured $Ce_6$ conjugate indicates internalized $Ce_6$. Cells incorporate occupied receptors and recycle free receptors back to their surface.

### Phototoxicity of bound, complexed and free $Ce_6$

The phototoxicity data on retinoblastoma cells treated with free, complexed and conjugated $Ce_6$ and light are presented in Figure 7. Consistent with the increased uptake, photosensitization was most

© *Cancer Research Campaign 1997*

**Figure 7** Phototoxicity to retinoblastoma cells of free $Ce_6$, mixed and covalently bound $Ce_6$–LDL. A fluence of 10 J $cm^{-2}$ with an irradiance of 50 mW $cm^{-2}$ was delivered at 660 nm. Different binding or mixing ratios from 1:10 to 1:1000 of LDL:$Ce_6$ were used. Cells were incubated with the complex conjugate for 2 h. Dark controls received $Ce_6$:LDL at a ratio of 50:1 without light exposure. Cell survival was measured by determination of mitochondrial activity using an MTT assay. The open bar shows $Ce_6$ phototoxicity. Cells were incubated with 4 nmol of $Ce_6$ for 2 h. Error bars are s.e.m. ($P$<0.01)

effective with conjugated $Ce_6$–LDL. Again, a ratio of 50:1 of $Ce_6$:LDL exhibited the highest phototoxicity. At this ratio, typically only 20% survival of cells was observed at a fluence of 10 J $cm^{-2}$, while $Ce_6$ in its free form showed no significant phototoxicity. The $Ce_6$–LDL complex showed 80–90% cell survival. None of the compounds showed dark toxicity.

### DISCUSSION

Photodynamic strategies are attractive owing to their relatively non-invasive nature and their potential for increased selectivity compared with conventional treatment modalities. For the treatment of retinoblastomas only under conditions in which these two aspects are fulfilled, PDT would be preferable to most established methods, such as external beam radiotherapy, plaque radiotherapy, cryotherapy and photocoagulation. Complications of radiation therapy, however, are known to be serious: induction of second cancers, retinopathy with visual loss, sunken globe, periorbital bone deformities and central nervous system abnormalities are well documented (Abramson et al, 1981; Sery, 1987; Shields et al, 1993). Therefore, alternative approaches, such as chemotherapy, are currently being discussed.

Targeted photochemotherapy should increase the ratio of tumour toxicity to normal tissue toxicity by causing damage primarily to target cells within a treatment site to which illumination is tightly confined. The most effective PS for PDT in vivo are believed to derive their tumour localization by binding to lipoproteins following systemic administration (Jori et al, 1990). In addition, Jori and Reddi (1990) and others (Mosley et al, 1981; Allison et al, 1994; Schmidt-Erfurth et al, 1994) have demonstrated that many photosensitizers target tumour cells more efficiently when precomplexed with LDL before administration in vivo. The motivation for this study was to carry the encouraging results of these previous investigations with PS–LDL complexes a step further and to investigate whether covalent linking of PS to LDL would provide an advantage over simple mixing of PS with LDL (complex), both in terms of PS uptake and phototoxicity. In addition, complex formation leaves open the possibility of the PS binding with other blood components that may not help tumour targeting. In addition, the molecules that may be complexed to LDL easily are limited by their hydrophilicity, since it is largely the hydrophobic forces of a molecule that make the complex formation possible (Jori, 1989).

$Ce_6$ was selected as a sensitizing agent for this study over haematoporphyrin derivative (HPD), which is the primary sensitizer used clinically (Weishaupt et al, 1976; Bruce, 1987; Sery et al, 1987; Tse et al, 1989). $Ce_6$ offers a high singlet oxygen quantum yield and reduced skin phototoxicity (Moan et al, 1987). Also, it is relatively less hydrophobic than HPD, so that the potential for using LDL as a carrier for less hydrophobic molecules could also be tested. $Ce_6$ was covalently bound to LDL by peptide bond formation catalysed by a carbodiimide reaction. Quantitative effectiveness of the binding procedure and qualitative preservation of the phototoxic potential of $Ce_6$ had previously been shown by coupling of the same compound to MAb and microspheres (Goff et al, 1991, 1994; Bachor et al, 1991*a*, *b*, *c*). LDL seemed to be an ideal carrier owing to the presence of significantly increased numbers of receptors on cells with a high mitotic index (Gal et al, 1981; Vitols et al, 1985). Since there are no data available on the distribution of LDL receptors on retinoblastoma cells, the conjugate was first tested in fibroblast cultures, for which expression and regulation of the LDL receptor had previously been characterized (Brown and Goldstein, 1975). The same group later demonstrated targeted phototoxicity using pyrene complexed to LDL in the same cell type (Anderson et al, 1978). Exactly how cells recognize and endocytose LDL in its native or modified form is not completely understood. Three mechanisms have been suggested. Firstly, the apoB/E receptor, which is present on all cells, is the main mechanism by which cells gain exogenous cholesterol and can be regulated up or down to attain cholesterol homeostasis. There is some evidence that malignant cells can up-regulate their expression of the apoB/E receptor to satisfy their increased demand for cholesterol as a result of their greater rate of synthesis of membrane lipids (Rudling et al, 1990), and forming complexes or conjugates of drugs with LDL has been proposed as a means of selectively targeting tumours (Vitols, 1991). Secondly, the scavenger receptor (SR) is present on cells of the monocyte–macrophage lineage. The SR recognizes a wide range of ligands, including LDL, which has had one-sixth (approximately 60) of its epsilon amino groups (Haberland et al, 1984) modified either by acetylation or acylation by lipid oxidation products from LDL oxidation (Parthasarathy, 1991). Thirdly, the apoB/E-mediated phagocytosis mechanism, present on macrophages, recognizes LDL, which has been less severely modified than described above (Tertov et al, 1989), and is thought to depend upon an increase in aggregation of the LDL particles (Khoo et al, 1988). Which, if any, of the above mechanisms was responsible for the increased efficacy of PDT with LDL conjugates in the present study is not clear. It may be that different mechanism(s) will be dominant in different cell types. Differences in the kinetics of $Ce_6$-conjugates in fibroblasts compared with retinoblastoma cells were evident at different temperatures in these series (Figures 4 and 5). Such information may then be used for designing appropriately modified LDL moieties.

© Cancer Research Campaign 1997

Any method of conjugation to a receptor-targeting moiety needs to preserve the receptor recognition capabilities of the targeting molecule. Experiments in this investigation demonstrated that this was the case for $Ce_6$–LDL ratios of up to 50:1. In these experiments, uptake of $Ce_6$–LDL conjugates by fibroblasts and retinoblastoma cells was about five times higher compared with free $Ce_6$ Higher binding ratios than 50:1 gave lower uptake of $Ce_6$, presumably because they compromised the affinity of the receptor recognition site, apoprotein B and E, where $Ce_6$ binding occurred on the LDL protein.

In the present study, increased uptake directly translated into enhanced phototoxicity in the same range as the uptake. Since chemical binding is known to cause changes in the molecular photophysics and, hence, in factors such as the singlet oxygen quantum yield of a sensitizer as well as its intracellular distribution (Bachor et al, 1991*c*), the proportional increase or decrease in cell kill is rarely predictable. Exposure to higher doses than 10 J $cm^{-2}$ might have increased the photocytotoxicity further; however, the borderline radiant exposure used appropriately demonstrated the differential effects of free, complexed and bound $Ce_6$. Although, as shown in Figure 3, the photosensitizer content of cells is similar for free and complexed $Ce_6$, the complex appeared to be slightly more phototoxic than the free $Ce_6$. This may be due to $Ce_6$-induced photosensitization of LDL, resulting in lipid peroxidation products, which may be toxic to cells (Brown and Goldstein, 1975; Maziere et al, 1990). Alternatively, the small difference in the uptake or the intracellular site of $Ce_6$ localization is responsible for the increased phototoxicity with the $Ce_6$–LDL complex.

The initial experiments with PDT and retinoblastoma cells were performed by Sery et al (1979). Two cell lines, Y79 and WERI-RB1, were photosensitized with HPD and white light, and the resulting photodynamic effects were documented. Cell suspensions were supplemented with HPD, incubated for different time intervals and subsequently irradiated in the presence of HPD. The highest phototoxicity was achieved when sensitization periods of cell–dye contact were 6 h or longer. This extended interval demonstrates the slow rate of non-specific accumulation of free photosensitizer. When the HPD was washed out of the incubation medium before irradiation, phototoxicity was significantly reduced, similar to our observations with free $Ce_6$. Apparently, free HPD was not firmly bound to cells or internalized, but loosely attached through non-specific interactions or diffusion, in a rapidly reversible processes. When sensitizer bound to LDL was used in our study, intensive washing did not reduce the phototoxicity of $Ce_6$–LDL, while free $Ce_6$ was easily removed from the cells owing to lack of receptor binding, consistent with Sery's experience. Furthermore, HPD at very high concentrations of 16.7 μM was required to achieve phototoxicity. Addition of serum to the incubation medium induced an inhibitory effect on HPD phototoxicity, which supports the important role of plasma factors for the delivery (and removal) of free sensitizers.

Winther (1989) studied the effect of PF on a retinoblastoma-like cell line, EXP-5, in vitro, after in vivo experiments with the same cell line exhibited a tumour response of only 33%. Their results indicated an extremely high sensitivity of retinoblastoma cells in vitro to photodynamic treatment. A directly proportional relation between light and PS dose and the level of phototoxicity was found. In our study, an increased intracellular sensitizer concentration led to an enhanced phototoxicity, with an increased PS uptake with the use of LDL carriers. Direct tumour cell targeting, therefore, may represent a realistic approach to increasing the efficient PS concentration in tumour tissue, since the administration of higher PS doses or an extension of the incubation interval has limitations in vivo.

The contrast in high in vitro toxicity and low in vivo effects observed by Winther (1989) is often explained by the influence of PDT-induced hypoxia (Henderson and Fingar, 1987). For most PS currently in experimental laboratory or clinical use, PDT induces vascular occlusion and local ischaemia early during irradiation. Lack of oxygen, a critical mediator of phototoxicity, then eliminates photochemical effects in the tumour cells. Hence, to achieve complete tumour cures, a delivery system that potentially leads to specific accumulation within tumour cells is essential. The timing of the irradiation (time point following PS administration) strongly influences the site of phototoxic damage (Zhou et al, 1988). When the LDL–PS complexes or conjugates are used to deliver the PS for in vivo PDT of tumours, the reaction of these species with endothelial cells must be considered, since this is the barrier through which the PS must pass if tumour cells are to be photosensitized. Endothelial cells express the classical apoB/E receptor (high affinity, low capacity) that leads to endocytosis via coated pits and vesicles to endosomes and then to lysosomes where the apoB is degraded and the cholesterol used in the cell. In addition, approximately 60% of the LDL taken up by the endothelium is transcytosed by non-coated plasmalemmal vesicles or caveolae (low affinity, high capacity) (Simionescu and Simionescu, 1991). Transcytosis is not saturable (Rutledge et al, 1990); in conditions of hyperbetalipoproteinaemia, large amounts of LDL can cross the endothelium (Vasile et al, 1983). Receptors for mildly oxidized LDL, specifically CD36 (Endenmann et al, 1993) have been found associated with caveolin in endothelium (Lisanti et al, 1994), but the binding mechanism for native LDL leading to transcytosis are presently unclear.

Endothelial cells do not express the type I and II scavenger receptors (Itakura et al, 1993) found on macrophages, which recognize acetyl–LDL and highly oxidized LDL (Naito et al, 1992), but nevertheless their interaction with acetyl–LDL is well known (Voyta, 1984). It seems likely that modified LDL is transcytosed (and possibly further oxidized) by endothelial cells in order for it to be endocytosed and degraded by macrophages and smooth muscle cells. The question arises as to what extent LDL is modified by formation of complexes or covalent conjugates with a PS? Although this issue was not investigated specifically in the present study, it may be an important factor in producing the enhanced phototoxicity with $Ce_6$–LDL conjugates. It has been suggested (Hamblin and Newman, 1994) that complex formation between PS and LDL in vivo modifies the behaviour of the LDL (perhaps with some oxidative event), so that the PS–LDL complex behaves similarly to mildly oxidized LDL, being transcytosed by the tumour microvasculature endothelium and accumulated in tumour-associated macrophages. Allison et al (1994) found that the photosensitizer BPD-MA was better delivered to tumours in vivo associated with native LDL than with acetylated LDL. When LDL-bound sensitizers are used, enhanced vascular effects are seen at early exposure, and direct tumour cell kill is mostly found after prolonged intervals, probably following the metabolic pathway of LDL (Zhou et al, 1988). Direct targeting of malignant cells with LDL (or other specific carriers) may decrease damage to physiological tissue by a differential tumoricidal effect. Additionally, side-effects could be reduced by administration of significantly less sensitizer. Lower dose and potentially more selective binding reduce cutaneous phototoxicity commonly seen with HPD when lipoproteins are used as carriers (Jori et al, 1990).

© Cancer Research Campaign 1997 

When we used LDL to deliver the PS, benzoporphyrin derivative mono-acid (BPD-MA), to treat an experimental melanoma in vivo, vascular occlusion subsequent to endothelial damage was a prominent feature following treatment soon after BPD-MA injection (Schmidt-Erfurth et al, 1994, 1995*a*, *b*). In addition, data (Schmidt-Erfurth et al, 1994) were also consistent with direct tumour cell cytotoxicity.

Binding of LDL–sensitizer conjugates to neovascular endothelium in addition to tumour cells has been attributed to receptor-mediated binding of LDL by endothelial cells (Vlodavsky et al, 1978). This binding to endothelial cells may provide additional targeting of tumour neovasculature, which would further enhance phototoxicity (Roberts and Hasan, 1992). The ability of LDL to pass microvascular barriers, particularly when the permeability is increased, as seen in intraocular and other tumours (Roberts and Hasan, 1993), provides a facilitated access of LDL-conjugates to neoplastic tissue.

In summary, this initial study demonstrates an advantage to using PS–LDL covalent conjugates. No attempt was made in these investigations to optimize the PDT parameters, nor was there any attempt to develop new techniques of conjugate synthesis or of exquisite purification or characterization of the conjugates. These initial encouraging results in the two cell lines tested warrant more rigorous studies and also suggest considering PDT with PS–LDL conjugates for intraocular and other malignancies. The use of carriers could potentially provide a direct and more specific targeting with increased efficiency and selectivity. Furthermore, it widens the repertoire of potential sensitizers based on their phototoxic potential, without the need of inherent selectivity or the ability to form complexes with LDL on the part of the photosensitizer.

### ACKNOWLEDGEMENTS

We thank Mr Michael Bamberg for preparation of the LDL, Dr Michael Hamblin for a critical review of the manuscript, Dr W G Roberts for helpful discussions and Coherent Inc. (Palo Alto) for the loan of the argon laser. This work was supported in part by the Office of Naval Research contract ONR N00014-94-1-0927 and a National Institutes of Health grant R01AR40352-04. Ursula Schmidt-Erfurth was a recipient of a fellowship from the German Research Council (Schm 836). Heyke Diddens received a travel grant from the German Research Council (Di309/1-1).

### REFERENCES

Abramson DH, Jeeb B and Ellsworth RM (1981) External beam radiation for retinoblastoma. *Bull NY Acad Med* **57**: 787–803

Allison BA, Pritchard PH, Richter AM and Levy JG (1990) The plasma distribution of benzoporphyrin derivative and the effects of plasma lipoproteins on its biodistribution. *Photochem Photobiol* **52**:501–507

Allison BA, Pritchard, PH and Levy JG (1994) Evidence for low-density lipoprotein receptor-mediated uptake of benzoporphyrin derivative. *Br J Cancer* **69**: 833–839

Anderson RGW, Vasile E, Mello RJ, Brown MS and Goldstein JL (1978) Immunocytochemical visualization of coated pits and vesicles in human fibroblasts: relation to low-density lipoprotein receptor distribution. *Cell* **15**: 919–933

Bachor R, Shea CR, Gillies R and Hasan T (1991*a*) Photosensitized destruction of human bladder carcinoma cells treated with chlorin $e_6$-conjugated microspheres. *Proc Natl Acad Sci USA* **88**: 1580–1584

Bachor R, Scholz M, Shea C and Hasan T (1991*b*) Mechanism of photosensitization by microsphere-bound chlorin $e_6$ in human bladder carcinoma cells. *J Urol* **51**: 4410–4414

Bachor R, Shea C and Hasan T (1991*c*) Free and conjugated chlorin $e_6$ in the photodynamic therapy of human bladder carcinoma cells. *J Urol* **146**: 1654–1658

Brown MS and Goldstein JL (1975) Regulation of the activity of the low density lipoprotein receptor in human fibroblasts. *Cell* **6**: 307–316

Bruce RA, JR (1987) Evaluation of hematoporphyrin photoradiation therapy techniques in the treatment of intraocular tumours. *Photochem Photobiol* **46**: 919–923

Dougherty, TJ (1987) Photosensitizers: therapy and detection of malignant tumours. *Photochem Photobiol* **45**: 879–889

Dougherty TJ (1989) Photodynamic therapy – new approaches (review). *Semin Surg Oncol* **5**: 6–16

Endenmann G, Stanton LW, Madden KS, Bryant CM, White RT and Protter AA (1993) CD36 is a receptor for oxidized low density lipoprotein. *J Biol Chem* **268**: 11811–11816

Gal D, Mcdonald PC, Porter JC and Simpson ER (1981) Cholesterol metabolism in cancer cells in monolayer culture. III. Low-density lipoprotein metabolism. *Int J Cancer* **28**: 315–319

Goff BA, Bamberg M and Hasan T (1991) Photoimmunotherapy of human ovarian carcinoma cells *ex vivo*. *Cancer Res* **51**: 4762–4767

Goff BA, Hermanto U, Rumbaugh J, Blake J, Bamberg M and Hasan, T (1994) Photoimmunotherapy and biodistribution with an OC125-chlorin immunoconjugate in an *in vivo* murine ovarian cancer model. *Br J Cancer* **70**: 474–480

Gomer CJ (1991) Preclinical examination of first and second generation photosensitizers used in photodynamic therapy. *Photochem Photobiol* **34**: 1093–1107

Haberland ME, Olch CL and Folgelman AM (1984) Role of lysines in mediating interaction of modified low density lipoproteins with the scavenger receptor of human monocyte macrophages. *J Biol Chem* **259**: 11305–11311

Hamblin MR and Newman EL (1994) Photosensitizer targeting in photodynamic therapy. II. Conjugates of haematoporphyrin with serum lipoproteins. *J Photochem Photobiol B* **26**: 147–157

Hasan T (1992) Photosensitizer delivery mediated by macromolecular carrier systems. In *Photodynamic Therapy: Basic Principles and Clinical Applications*. Henderson B and Dougherty T (eds). Marcel Dekker, 187–200

Hasan T and Parrish JA (1996) Photodynamic therapy of cancer. In *Cancer Medicine* 4th edn. Holland JF (ed.). Williams and Wilkins Vol 1, Chpt 50, 739–751

Henderson BW and Dougherty TJ (1992) How does PDT work? *Photochem Photobiol* **55**: 145–157

Henderson BW and Fingar VJ (1987) Relationship of tumour hypoxia and response to photodynamic treatment in an experimental mouse tumour. *Cancer Res* **47**: 3110–3114

Itakura H, Matsumoto A, Asaoka H and Kodama T (1993) Structure and function of the scavenger receptor. *Nippon Rinsho* **51**: 1083–1091

Jiang FN, Liu DJ, Neyndorff H, Chester M, Jiang S and Levy JG (1991) Photodynamic killing of human squamous cell carcinoma cells using a monoclonal antibody–photosensitizer conjugate. *J Natl Cancer Inst* **83**: 1218–1225

Jori G (1989) *In vivo* transport and pharmacokinetic behaviour of tumour photosensitizers. In *Photosensitizing Compounds: their Chemistry, Biology and Clinical Use*. Ciba foundation Symposium 146 John Wiley and Sons: Chichester, UK.

Jori G (1990) Factors controlling the selectivity and efficiency of tumour damage of photodynamic therapy. *Lasers Med Sci* **5**: 115–120

Jori G, Beltramini M, Reddi E, Pagnan A, Tomio L and Tsanov T (1984) Evidence for a major role of plasma lipoproteins as hematoporphyrin carriers *in vivo*. *Cancer Lett* **24**: 291–297

Jori G and Reddi E (1990) Strategies for tumour targeting by photodynamic sensitizers. In *Photodynamic Therapy of Neoplastic Disease*. Kessel D (ed.). CRC Press: Boca Raton, FL. 117–130

Khoo JC, Miller E, Mcloughlin P and Steinberg D (1988) Enhanced macrophage uptake of low density lipoprotein after self-aggregation. *Arteriosclerosis* **8**: 348–358

Larson E, Howlett B and Jagendorf A (1986) Artificial reductant enhancement of the Lowry method for protein determination. *Anal Biochem* **155**: 243–248

Lisanti MP, Scherer PE, Vidugieriene J, Tang Z, Hermanowski-Vosatka A, Tu YH, Cook RF and Sargiacomo M (1994) Characterization of caveolin-rich membrane domains isolated from an endothelial-rich source: implications for human disease. *J Cell Biol.* **126**: 111–126

© Cancer Research Campaign 1997

Maziere JC, Santus R, Morliere P, Reyftmann JP, Candide C, Mora L, Salmon S, Maziere C, Gatt S and Dubertret L (1990) Cellular uptake and photosensitizing properties of anticancer porphyrins in cell membranes and low and high density lipoproteins (review). *J Photochem Photobiol* **6**: 61–68

Mew D, Wat CK, Towers GHN and Levy JG (1983) Photoimmunotherapy: treatment of animal tumours with tumour-specific monoclonal antibody–hematoporphyrin conjugates. *J Immunol* **130**: 1473–1477

Moan J, Peng Q, Evensen JF, Berg K, Western A and Remington C (1987) Photosensitizing efficiencies tumour and cellular uptake of different photosensitizing drugs relevant for photodynamic therapy of cancer. *Photochem Photobiol* **46**: 713–721

Mosley ST, Goldstein JL, Brown MS, Falck JR and Anderson RGW (1981) Targeted killing of cultured cells by receptor-dependent photosensitization. *Proc Natl Acad Sci USA* **78**: 5217–5721

Murphree AL, Cote M and Gomer CJ (1987) The evolution of photodynamic therapy techniques in the treatment of intraocular tumours. *Photochem Photobiol* **46**: 919–923

Naito M, Suzuki H, Mori T, Matsumoto A, Kodama T and Takahashi K (1992) Coexpression of type I and type II human macrophage scavenger receptors in macrophages of various organs and foam cells in atherosclerotic lesions. *Am J Pathol* **141**: 591–599

Oseroff AR, Ohuoha D, Hasan T, Bommer JC and Yarmush ML (1986) Antibody-targeted photolysis: selective photodestruction of human T-cell leukemia cells using monoclonal antibody–chlorin $e_6$ conjugates. *Proc Natl Acad Sci USA* **83**: 8744–8748

Parthasarathy S (1991) Novel atherogenic, oxidative modification of low-density lipoprotein. *Diabetes Metab Rev* **7**: 163–171

Roberts WG and Hasan T (1992) Role of neovasculature and vascular permeability on the tumour retention of photodynamic agents. *Cancer Res* **52**: 924–930

Roberts WG and Hasan T (1993) tumour-secreted vascular permeability factor influences photosensitizer uptake. *Cancer Res* **53**: 1–5

Rudel U, Lee JA, Morris MD and Fells JM (1974) Characterization of plasma lipoproteins separated and purified by agarose-column chromatography. *Biochem J* **139**: 89–95

Rudling MJ, Angelin B, Peterson CO and Collins VP (1990) Low density lipoprotein receptor activity in human intracranial tumours and its relation to the cholesterol requirement. *Cancer Res* **50**: 483–487

Rutledge JC, Curry F-RE, Lenoc JF and Davis PA (1990) Low density lipoprotein transport across a microvascular endothelial barrier after permeability is increased. *Circ Res* **66**: 486–494

Sargent JM and Taylor CG (1989) Appraisal of the MII assay as a rapid test of chemosensitivity in acute haircell leukemia. *Br J Cancer* **60**: 206–210

Schmidt-Erfurth U, Bauman W, Gragoudas E, Flotte TJ, Michaud NA, Birngruber R and Hasan T (1994) Photodynamic therapy of experimental choroidal melanoma using lipoprotein-delivered benzoporphyrin. *Ophthalmology* **101**: 89–99

Schmidt-Erfurth U, Flotte TJ, Gragoudas ES, Schomacker K, Birngruber R and Hasan T (1995*a*) Benzoporphyrin–Lipoprotein mediated photodestruction of intraocular tumours. *Exp Eye Res* **62:** (in press)

Schmidt-Erfurth U, Hasan T, Schomacker K, Flotte T and Birngruber R (1995*b*) *In vivo* uptake of liposomal benzoporphyrin derivative and photothrombosis in experimental corneal neovascularization. *Lasers Surg Med* **17**: 178–188

Sery T (1979) Photodynamic killing of retinoblastoma cells with hematoporphyrin and light. *Cancer Res* **39**, 90–100

Sery TW, Shields JA, Augsburger JJ and Shah HG (1987) Photodynamic therapy of human ocular cancer. *Ophthal Surg* **18**: 413–418

Shields CL, Shield JA, De Potter P, Muelli S, Hernandes C, Brady LW, and Cater JR, (1993) Plaque radiotherapy in the management of retinoblastoma. *Ophthalmology* **100**: 216–224

Simionescu, N and Simionescu M (1991) Cellular interactions of lipoproteins with the vascular endothelium: endocytosis and transcytosis. *Targeted Diagn Ther* **5**: 45–95

Suits AG, Chait A, Aviram M and Heinecke JW (1989) Phagocytosis of aggregated lipoprotein by macrophages: low density lipoprotein receptor-dependent foam-cell formation. *Proc Natl Acad Sci USA* **86**: 2713–2717

Tertov VV, Sobenin IA, Gabbasov ZA, Popov EG and Orekhov AN (1989) Lipoprotein aggregation as an essential condition of intracellular lipid accumulation caused by modified low density lipoproteins. *Biochem Biophys Res Commun* **163**: 489–494

Tse DT, Dutton JJ and Weingeist TA (1989) Hematoporphyrin photoradiation therapy for intraocular and orbital malignant melanoma. *Arch Ophthalmol* **102**: 833–838

Van Hillegersberg R, Kort WJ and Wilson JH (1994) Current status of photodynamic therapy in oncology (Review). *Drugs* **48**: 510–527

Vasile E, Simionescu M and Simionescu N (1983) Visualization of the binding, endocytosis, and transcytosis of low-density lipoprotein in the arterial endothelium *in situ*. J Cell Biol **96**: 1677–1689

Vitols S (1991) Uptake of low-density lipoprotein by malignant cells – possible therapeutic applications. *Cancer Cells* **3**, 488–495

Vitols S, Gahrton G, Bjorkhol M and Peterson C (1985) Hypocholesterolemia in malignancy due to elevated LDL receptor activity in tumour cells. *Lancet* **2**: 1150–1154

Vlodavsky I, Fielding PE, Fielding CJ and Gospodarowicz D (1978) Role of contact inhibition in the regulation of receptor-mediated uptake of low density lipoprotein in cultured vascular endothelial cells. *Proc Natl Acad Sci USA* **75**: 356–360

Voyta JC, Via DP, Butterfield CE and Zetter BR (1984) Identification and isolation of endothelial cells based on their increased uptake of aetylated low-density lipoprotein. *J Cell Biol* **99**: 2034–2040

Weishaupt UR, Gomer CJ and Dougherty TJ (1976) Identification of singlet oxygen as the cytotoxic agent in photoinactivation of a murine tumour. *Cancer Res* **36**: 2326–2329

Winther J (1989) Photodynamic therapy effect in an intraocular retinoblastoma-like tumour assessed by an *in vivo* to *in vitro* colony forming assay. *Br J Cancer* **59**: 869–872

Zhou C, Milanesi C and Jori G (1988) An ultrastructural comparative evaluation of tumours photosensitized by porphyrins administered in aqueous solution, bound to liposomes or to lipoproteins. *Photochem Photobiol* **48**: 487–492

© Cancer Research Campaign 1997